# Association between Backward Walking and Cognition in Parkinson Disease: A Systematic Review

**DOI:** 10.3390/ijerph191912810

**Published:** 2022-10-06

**Authors:** Mathias Baptiste Correno, Clint Hansen, Matthias Chardon, Tracy Milane, Edoardo Bianchini, Nicolas Vuillerme

**Affiliations:** 1 AGEIS, Université Grenoble Alpes, 38000 Grenoble, France; 2 Department of Neurology, Kiel University, UKSH Campus Kiel, Arnold-Heller-Str. 3, Haus D, 24105 Kiel, Germany; 3 Department of Neuroscience, Mental Health and Sensory Organs (NESMOS), Sapienza University of Rome, 00185 Rome, Italy; 4 LabCom Telecom4Health, Orange Labs & Université Grenoble Alpes, CNRS, Inria, Grenoble INP-UGA, 38000 Grenoble, France; 5 Institut Universitaire de France, 75005 Paris, France

**Keywords:** gait impairments, dynamic balance, cognition, clinical tool, Parkinson’s disease

## Abstract

Backward walking often occurs in everyday life. It is more complex than forward walking and is associated with decreased coordination. However, it is unclear if a reduced backward walking performance is associated with impaired cognition. This could be particularly relevant as gait and cognitive deficits commonly occur in Parkinson’s disease. The objective of this systematic review was to synthesize the evidence on the association between backward walking and cognition in persons with Parkinson’s disease. The electronic databases PubMed and Web of Science were systematically searched, and the quality of eligible studies was assessed. Two studies met the inclusion criteria, but study protocols, investigated population, and outcome measures differed substantially. One study showed lower backward walking speed in patients with Parkinson’s disease with poorer attention test performances. The second study showed a weak correlation between executive cognitive functions and backward walking speed. Given the low number of studies, the heterogenous study design, and the inconsistent results, the present review highlights the need to further investigate the association between backward walking and cognition in patients with Parkinson’s disease.

## 1. Introduction

Parkinson’s Disease (PD) is a neurodegenerative disease characterized by both motor and non-motor symptoms. Among non-motor symptoms, cognitive impairment is relatively common in people with PD. People with PD exhibit a quicker decline in several cognitive functions (i.e., especially in executive, attentional, and visuospatial domains) when compared to age-matched healthy adults [1,2]. Gait and balance impairments are also common deficits in PD and significantly impact the quality of life of patients with reduced functional independence and an increased incidence of falls [3]. Although pharmacologic treatment is very effective in improving PD-related motor symptoms [4], gait and balance disorders are usually less responsive to dopaminergic therapy [5,6]. Gait has two primary phases; the stance and swing phases. The stance phase consists of the time the foot is in contact with the ground, and the swing phase consists of the time the foot is in the air. Parkinsonian gait is characterized by a reduced swing phase, slower walking speed, and shorter stride length [7,8]. Stride length is the distance between two initial contacts of one foot. Step frequency/cadence might remain unchanged compared to a healthy gait [9,10]. However, step frequency can be considered a compensatory mechanism for a reduced stride length [10] while maintaining the same walking speed [11].

In everyday life, we walk in multiple directions, including side-stepping and backward walking. These tasks often occur without notice, but when purposely walking backward, the task seems complicated, showcasing decreased coordination [12]. Further, backward walking speed decreases, and the risk of falls increases with age [13,14]. Hence backward walking is often used in physiotherapy to improve gait characteristics and mobility of the lower extremities. Frequent backward walking improves knee, hip, and ankle range of motion, strength, and coordination in children with cerebral palsy [15,16] and individuals with neurological and musculoskeletal pathologies [17].

According to Hackney et al. [18], patients with PD have more significant deficits in backward walking than forward walking and a reduction in backward walking speed [17,19]. The authors proposed that the neural correlates implemented for backward walking could be impacted earlier in people with PD compared to controls. Gait relies on multi-sensory information and the descending pathways from the brainstem to the spinal cord. In his review, Takakusaki [20] highlighted that the basal ganglia and cerebellum affect the automatic and cognitive processes of walking. When walking in unfamiliar circumstances, the cognitive load of postural control increases and impairments in cognitive function may result in falling. During backward walking, Hackney et al. [17] showed significantly slower, shorter strides, lower swing, and higher double support and stance percentages in patients with PD compared to controls. People with PD also require more proprioception [19] and attention [21] while walking backward. Those with mild to moderate PD usually show impaired forward and backward walking, but differences between those with and without PD are more pronounced in backward walking [18].

The evidence could indicate that cognitive impairment may be an essential factor regarding backward walking performance. The relationship between forward walking and cognition is known, and the reduced executive performance observed in PD has been linked to gait impairment. However, evidence is scarce to date about the relationship between backward walking and cognitive function in PD. Hence, this systematic review aimed to synthesize the published studies that related backward walking to cognitive difficulties in PD patients, highlighting its potential clinical use.

## 2. Materials and Methods

The protocol registration of this systematic review happened in the prospective register of systematic reviews (CRD42021274763). The Preferred Reporting Items for Systematic Reviews and Meta-Analysis guidelines were met [22], see Appendix A, Table A2.

### 2.1. Search Strategy

The two electronic databases, PubMed and Web of Science, were systematically searched with no limitation on publication date. A first preliminary search was conducted in March 2021, and it was repeated in March 2022 before the final review. Keywords related to (1) the population, (2) the walking direction, and (3) the cognition state. The search strategy included a combination of keywords, using the Boolean operators “AND” and “OR”. The first category focused the search on patients with PD and included terms such as “idiopathic Parkinson’s Disease”, “Lewy Body Parkinson Disease”, “Primary Parkinsonism”, “Idiopathic Parkinson Disease”, or “Parkinson Disease”. The second category focused on studies reporting backward walking, including terms such as “backwards walking”, “backward walking”, “backward gait”, “backward locomotion”, “backwards locomotion”, “retrowalking”, or “retro-walking”. Finally, the last category specified the state of cognition to correlate with backward walking. It comprised all terms relative to cognition: “cognition”, “cognitive”, “mental”, “dementia”, or “attention”. These three categories of keywords were combined for the final search as follows: (1) “AND” (2) “AND” (3). Search fields were restricted to the abstract, title, and keywords.

### 2.2. Selection of Articles

Inclusion criteria:

- original peer-reviewed scientific journal articles on humans published in English, French, or German;
- studies reporting both backward walking and cognitive variables to assess the relationship between these variables. Only cross-sectional studies and longitudinal studies were included.

Case reports, abstracts, editorials, letters to the editor, case studies, reviews, or meta-analyses were excluded. Studies not examining the association between backward walking and cognitive function in PD were also excluded.

### 2.3. Quality Assessment

The aim of this review was not to evaluate the effect of an intervention. A Study Quality Assessment Tool (see Appendix A, Table A1) was applied to assess the methodological quality of the included studies [23]. Two independent reviewers appraised the quality assessment. Disagreements between the first and the second were resolved through discussion.

### 2.4. Data Extraction

Two independent reviewers screened the titles and abstracts of all studies to identify potentially relevant articles and removed duplicates. Full texts of all studies that met the inclusion criteria were reviewed. Disagreements between the two reviewers were resolved through consensus through discussion and with a third reviewer.

Five characteristics were extracted from the retrieved articles: (1) study characteristics, (2) participant characteristics, (3) measure of backward walking, (4) measure of cognitive function, and (5) main findings of the association between backward walking performance and cognitive function in PD populations. Reviewers were not blinded to the authors or journals when extracting data.

## 3. Results

### 3.1. Study Selection

**Figure 1:**
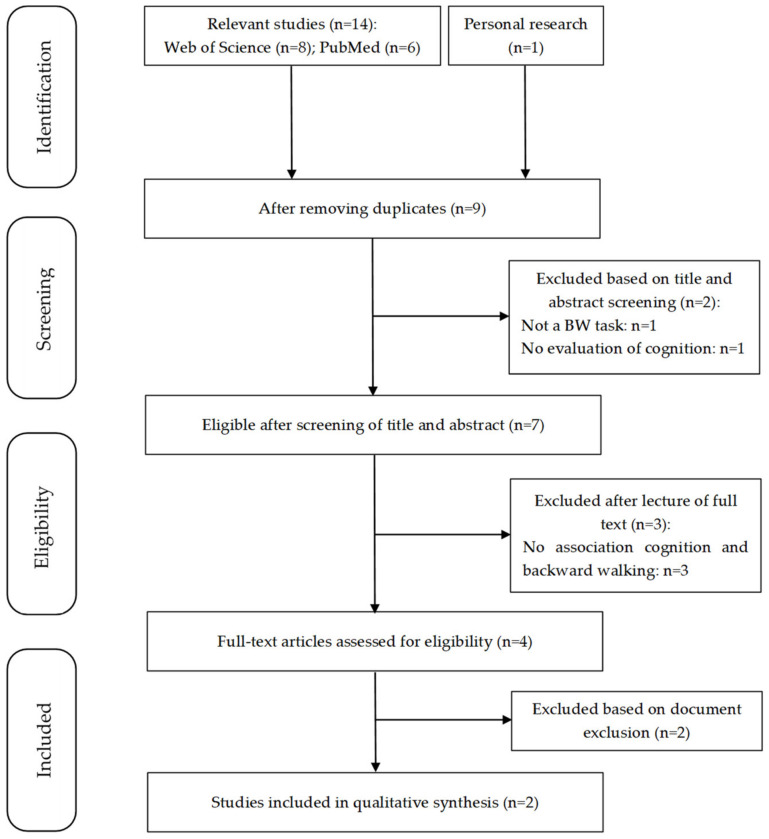
Flowchart of study selection. A total of two studies were included according to eligible criteria (n = number of studies, BW = backward walking).

The search strategy yielded 14 potentially relevant studies, and one record was identified from personal research, resulting in 15 studies. The flowchart (Figure 1) describes the selection process. After removing duplicates (*n* = 6), nine studies remained. After screening titles and abstracts, two studies were excluded, and seven were reviewed. After full-text reading, two studies fulfilled the eligibility criteria [21,24].

The quality assessment of the included studies showed “good” scores [21,24]. Details of quality assessment are available in Supplementary Material Table S1.

### 3.2. Study Characteristics

The two articles are cross-sectional studies [21,24]. Christofoletti et al. [24] investigated a cohort of 114 participants with PD, and Tseng et al. [21] compared a PD population to a control group [21]. The main characteristics are summarized in Table 1.

### 3.3. Participant Characteristics

The studies involved a total of 136 PD patients and 42 healthy controls. The sample size was *n* = 54.3 ± 42.2, ranging from 22 [21] to 114 [24] participants with PD. The mean PD patient’s age was 67.3 ± 2.3 years, presenting a range from 64.9 [24] to 70.50 years [21]. The repartition of male/female patients is balanced; however, Christofoletti et al. [24] did not indicate the repartition of gender. Among the two included studies, only Tseng et al. [21] reported the Body Mass Index (23.0 ± 4.3). The mean disease duration was 5.7 ± 0.7 years, ranging from 5.1 [21] to 5.4 years [24]. Demographics and disease characteristics are presented in Table 1.

### 3.4. Backward Walking and Study Design

Walking speed was evaluated in both articles [21,24]. Stride length, cadence, and swing phase were the main outcomes in Tseng et al. [21]. Gait parameters such as gait speed, stride length, cadence, and swing phase were used to describe forward walking and backward walking in individuals with PD.

In both protocols, walking speed was defined as a comfortable self-selected pace. Christofoletti et al. [24] offered a cognitive dual task for both forward and backward walking. Tseng et al. [21] analyzed gait in participants wearing shoes Christofoletti et al. [24] measured gait parameters during barefoot walking. Christofoletti et al. [24] collected three trials for each condition, while Tseng et al. [21] collected one trial per condition. The measurement device used to measure gait parameters was an instrumented GaitRITE electronic system of 5 m [19,22]. Rest was not reported consistently.

### 3.5. Measures of Disease Severity and Cognitive Function

There are few tests in common between the included studies. Both studies [21,24] used the Hoehn and Yahr (H&Y) stage to describe PD severity [25], with a mean H&Y stage of 2.3 for most participants [24] and a lower H&Y stage for one cohort [21]. Motor severity was also measured with the Movement Disorder Society Unified Parkinson’s Disease Rating Scale Part III (MDS-UPDRS-III) [26] in Christofoletti et al. [24] with overall MDS-UPDRS-III scores of 34.8 ± 10.4. The differences in the obtained scores suggest a less advanced state in the disease for participants of Tseng et al. [21].

In terms of cognitive scales, the Mini-Mental State Examination (MMSE) [27] was used in both studies [19,22], with scores of 28.6 ± 1.4 and 26.82 ± 3.11. Tseng et al. [21] used the ‘‘divided attention’’ subtest of the Test Battery for Attention Performance (version 2.0), including the accuracy and reaction time of a visual, auditory, and dual-task (visual and auditory) test. The performance of the dual-task test was used to dichotomize the participants into those with low and good attention capability to compare the two groups. Christofoletti [24] investigated execute functions using the Trail Making Test (score computed as Part B minus Part A), a Color–Word Interference test (score calculated as inhibition time minus color naming time) and a Verbal Fluency test.

### 3.6. Main Findings of the Association between Backward Walking Performance and Cognitive Function in PD Populations

Tseng et al. [21] compared gait parameters from forward and backward walking to the level of attention Test Battery for Attention Performance (TAP; version 2.0). They found that PD with lower attention, based on visual-auditory dual-task test performance, presented worse gait deficits in backward walking. All PD patients showed worse backward walking patterns compared to controls, even though the MMSE scores were comparable with healthy controls in the good attention PD group. PD and controls showed slower speeds in backward walking compared to forward walking.

Christofoletti et al. [24] reported a weak correlation between backward walking speed and the performance of both verbal fluency (R = 0.334) and the Color–Word Interference Test (R = −0.291), with lower test performance associated with slower speed. When included in the regression model, executive functions solely explained only 3.0% of the variance in gait speed in forward walking. Table 2 shows a summary of the interaction effects between cognitive function and walking direction.

## 4. Discussion

Two studies, including a total of 136 PD patients and 42 healthy controls, were included. Both studies reported measures of global and executive functions, even though different tests were used. The reported results showed conflicting results on the interaction between backward walking and cognitive performances in PD patients.

The study from Tseng et al. [21], showed a difference in backward walking speed between PD patients (with good and low attention test performance) with a slower backward walking speed in the low attention test performance group. Although the MMSE score was lower in poor-attention PD patients, it was not significantly different between the two groups. These results suggest a domain-specific association between cognition and backward walking.

In forward walking, the association between gait parameters and specific cognition domains, namely executive functions, has already been reported [28]. To this extent, the results from Tseng et al. [24] could suggest that the MMSE, a measure of global cognitive functioning, could not be sensitive enough to show an association with backward gait parameters. However, cognitive measures were used to dichotomize groups, and no direct correlation analyses were conducted to assess the association between them and backward walking speed.

Conversely, the results from Christofoletti et al. [24] showed only a weak correlation between executive functions and backward walking speed. The impact of executive functions on speed variance was significant only for forward walking and by a negligible entity (3%). The authors explained the difference between previous studies (expected 6–10% of explained variance) and their results based on different cognitive tests and walking protocols.

Considering the different test strategies, comparing the two populations on their baseline level of executive function performance is impossible. However, H&Y staging and MMSE test suggest that Tseng’s PD population was older, with lower disease severity and global cognitive function. In Christofoletti et al. [24], the relatively high cognitive status and low age could have led to a ceiling effect, dampening the eventual association between cognition and backward walking performance. This could, at least, partially explain the discrepancies between the two studies.

In PD, an alteration of several cognitive domains, including executive functions, could also be demonstrated in early disease stages [29] and even in patients with a normal MMSE score [30]. A score of 24 defines cognitive impairment. However, some studies suggested a conservative value of 27 to detect mild cognitive impairment in PD [31]. Therefore, the MMSE reported score by Tseng et al. [21] could suggest that the average cognitive level of enrolled PD patients is at the limit of mild cognitive impairment.

Moreover, the cognitive executive burden of walking increases with aging, as demonstrated by functional imaging studies [32], and the older age of patients in Tseng et al. [21] could affect the results. Finally, both studies adopted a very different approach to evaluating the association between cognitive functions and backward walking. Tseng et al. [21] compared two sub-populations of PD based on a principal component analysis of the performance of the auditory-visual dual-task test. Christofoletti et al. [24], on the other hand, used a more rigorous approach directly assessing the association between cognitive tests and backward walking speed.

When comparing forward and backward walking to the medication state, fewer gait parameters improved in response to dopaminergic therapy. This suggests that backward walking may provide additive information on mobility, especially in a clinical setting [33]. Just as in forward walking, some gait parameters in backward walking can be easily measured and provide relevant information: backward walking speed is a sensitive clinical marker of fall risk in PD and MS [13,34]. Edwards et al. [34] showed that backward walking speed is the strongest and unique descriptor of retrospective falls reported in MS patients. In addition, the age-related decline in walking appears to differ with direction and is more pronounced in backward walking [35].

While the low numbers of included studies suggest the innovative character and the potential of backward walking for clinical tests and diagnosis, the small panel of included studies (*N* = 2) is explained by the selection criteria associating cognitive function and gait parameters. The use of backward walking in people with PD is mainly reported in physiotherapy programs for individuals with neurological disorders [16,17] or rehabilitation programs in post-stroke patients [36], chronic incomplete spinal cord injury [37], and even in diabetic peripheral neuropathy patients [38].

## 5. Conclusions

This systematic review documents the relationship between cognitive performances in PD and backward walking. Two studies, heterogeneous for investigated population, outcome measures, and study protocol, show inconsistent results. Given the evidence of the association between forward walking and executive functions, particularly in PD patients, the present review highlights the need for further research to clarify the impact of cognitive performance on backward walking.

## Figures and Tables

**Table 1:** Characteristics of persons with Parkinson’s disease (PD) and summary of walking conditions used in the included studies.

Study	Tseng et al., 2012 [21]	Christofoletti et al., 2016 [24]
Number of PD patients	22	114
Number of controls	42	-
Age (ys) (mean ± SD)	70.5 ± 8.8	66.6 ± 9.4
Gender Male/Female (*n*, %)	11(50)/11(50)	-
BMI in kg/m^2^ (mean ± SD)	23.00 ± 4.31	-
Disease duration (ys) (mean ± SD)	5.09 ± 4.87	5.4 ± 4.4
H&Y stage (mean ± SD)	1.5 ± 0.5	2.4 ± 0.4
MDS-UPDRS-III (mean ± SD)	-	34.8 ± 10.4
MMSE (mean ± SD)	26.82 ± 3.11	28.6 ± 1.4
Training trials	Yes, 5 in either each condition	-
Walking speed	Preferred pace	Preferred speed
Experimental conditions	*N* = 2 Forward/backward walking	*N* = 4 Forward/forward walking with a cognitive task (phonemic listing task using a different letter), forward walking fast as possible, backward walking
Number of trials per condition	One trial for each condition	3
Walking distance	5.0 m	4.8 m
Collection of gait parameters	5 m Instrumented GAITRite walkway	4.8 m GAITRite instrumented walkway
Rest	Yes. Rest sitting between the audition trial and formal test.	-
Order randomized	Not reported	Yes
Other information	Barefoot	-

BMI = Body Mass Index. H&Y = Hoehn and Yahr. MDS-UPDRS-III = Movement Disorder Society-revised version of the motor part of the Unified Parkinson Disease Rating Scale. MMSE = Mini-Mental State Examination.

**Table 2:** Summary of the interaction effect between cognitive function and walking direction.

Authors	Statistical Analysis	Effect of BW on Gait Parameters
Tseng et al., 2012 [21]	Post-hoc test	Speed: PD-P (1): FW: 62.98; BW: 32.69; ↓ 48.09% PD-B (2): FW: 98.78; BW: 63.63; ↓ 35.58% HC-P (3): FW: 86.67; BW: 54.86; ↓ 36.70% HC-B (4): FW: 109.06; BW: 84.65; ↓ 22.38% Interactions: -FW: 1 < 2, 1 < 3, 1 < 4, 3 < 4 -BW: 1 < 2, 1 < 3, 1 < 4, 2 < 4, 3 < 4 Swing phase: PD-P (1): FW: 33.15; BW: 30.36; ↓ 8.42% PD-B (2): FW: 36.65; BW: 34.8; ↓ 5.05% HC-P (3): FW: 36.66; BW: 33.68; ↓ 8.13% HC-B (4): FW: 37.69; BW: 36.82; ↓ 2.31% Interactions: -FW: 1 < 2, 1 < 3, 1 < 4 -BW: 1 < 2, 1 < 3, 1 < 4, 3 < 4 Stride length: PD-P (1): FW: 78.56; BW: 47.87; ↓ 39.07% PD-B (2): FW: 110.85; BW: 75.15; ↓ 32.21% HC-P (3): FW: 102.09; BW: 66.95; ↓ 34.42% HC-B (4): FW: 120.13; BW: 96.96; ↓ 19.29% Interactions: -FW: 1 < 2, 1 < 3, 1 < 4, 3 < 4 -BW: 1 < 2, 1 < 3, 1 < 4, 2 < 4, 3 < 4
Christofoletti et al., 2016 [24]	Pearson for parametric variables Spearman for non-parametric variables Regression coefficients	Direction: BW: −0.415 * MDS-UPDRS III: FW: −0.443 *; BW: −0.391 * MDS-UPDRS IV: FW: −0.069; BW: 0.068 Mini-BESTest: FW: 0.664 *; BW: 0.685 * CWIT: FW: −0.380 *; BW: −0.291 * VF: FW: 0.336 *; BW: 0.334 *

MDS-UPDRS = Movement Disorders of UPDRS. UPDRS = Unified Parkinson Disease Rating Scale. Mini-BESTest = Mini-Balance Evaluation Systems Test. CWIT = Color–Word Interference test. VF = Verbal Fluency. FW = forward walking. BW = backward walking. PD-B = Parkinson’s patients with better attention capability. PD-P = Parkinson’s patients with poorer attention capability. HC-B = healthy controls with better attention capability. HC-P = healthy controls with poorer attention capability. ↓ = decline. * Significant results at *p* < 0.05.

## Data Availability

Not applicable.

## Supplementary Materials

The following supporting information can be downloaded at: https://www.mdpi.com/article/10.3390/ijerph191912810/s1. Table S1: Bias Assessment of the included studies.

## Appendix A

The aim of this review was not to evaluate the effect of an intervention, thereby we did not use a risk of bias assessment. A Study Quality Assessment Tool (see Supplementary Table S1) was applied in order to assess the methodological quality of the included studies [21].

**Table A1:** Summary of walking conditions used in the included studies.

Criteria	Tseng and Yuan, 2012 [21]	Christofoletti et al., 2016 [24]
1. Was the research question or objective in this paper clearly stated?	Y	Y
2. Was the study population clearly specified and defined?	Y	Y
3. Was the participation rate of eligible persons at least 50%?	Y	Y
4. Were all the subjects selected or recruited from the same or similar population? Were inclusion and exclusion criteria for being in the study prespecified and applied uniformly to all participants?	Y	Y
5. Was a sample size justification, power description, or variance and effect estimates provided?	N	Y
6. For the analyses in this paper, were the exposure(s) of interest measured prior to the outcome(s) being measured?	Y	Y
7. Was the timeframe sufficient so that one could reasonably expect to see an association between exposure and outcome if it existed?	NA	NA
8. For exposures that can vary in amount or level, did the study examine different levels of the exposure as related to the outcome (e.g., categories of exposure, or exposure measured as continuous variable)?	N	Y
9. Were the exposure measures (independent variables) clearly defined, valid, reliable, and implemented consistently across all study participants?	Y	NA
10. Was the exposure(s) assessed more than once over time?	NA	NA
11. Were the outcome measures (dependent variables) clearly defined, valid, reliable, and implemented consistently across all study participants?	Y	Y
12. Were the outcome assessors blinded to the exposure status of participants?	NR	NR
13. Was loss to follow-up after baseline 20% or less?	NA	NA
14. Were key potential confounding variables measured and adjusted statistically for their impact on the relationship between exposure(s) and outcome(s)?	Y	Y
Quality Rating	Good	Good

**Table A2:** PRISMA Checklist [22]. The Preferred Reporting Items for Systematic Reviews and Meta-Analysis (PRISMA [22]) is a 27-item checklist to cover all main sections of the manuscript, including title, abstract, introduction, methods, results, discussion, and funding. Color codes are just to highlight the main sections.

Section and Topic	Item #	Checklist Item	Location where Item Is Reported
**TITLE**			
Title	1	Identify the report as a systematic review.	3
**ABSTRACT**			
Abstract	2	See the PRISMA 2020 for Abstracts checklist.	15–28
**INTRODUCTION**			
Rationale	3	Describe the rationale for the review in the context of existing knowledge.	32–45
Objectives	4	Provide an explicit statement of the objective(s) or question(s) the review addresses.	77–79
**METHODS**			
Eligibility criteria	5	Specify the inclusion and exclusion criteria for the review and how studies were grouped for the syntheses.	103–111
Information sources	6	Specify all databases, registers, websites, organisations, reference lists and other sources searched or consulted to identify studies. Specify the date when each source was last searched or consulted.	86–89
Search strategy	7	Present the full search strategies for all databases, registers and websites, including any filters and limits used.	86–101
Selection process	8	Specify the methods used to decide whether a study met the inclusion criteria of the review, including how many reviewers screened each record and each report retrieved, whether they worked independently, and if applicable, details of automation tools used in the process.	112–114
Data collection process	9	Specify the methods used to collect data from reports, including how many reviewers collected data from each report, whether they worked independently, any processes for obtaining or confirming data from study investigators, and if applicable, details of automation tools used in the process.	122–133
Data items	10a	List and define all outcomes for which data were sought. Specify whether all results that were compatible with each outcome domain in each study were sought (e.g., for all measures, time points, analyses), and if not, the methods used to decide which results to collect.	127–131
	10b	List and define all other variables for which data were sought (e.g., participant and intervention characteristics, funding sources). Describe any assumptions made about any missing or unclear information.	N/A
Study risk of bias assessment	11	Specify the methods used to assess risk of bias in the included studies, including details of the tool(s) used, how many reviewers assessed each study and whether they worked independently, and if applicable, details of automation tools used in the process.	116–120
Effect measures	12	Specify for each outcome the effect measure(s) (e.g., risk ratio, mean difference) used in the synthesis or presentation of results.	N/A
Synthesis methods	13a	Describe the processes used to decide which studies were eligible for each synthesis (e.g., tabulating the study intervention characteristics and comparing against the planned groups for each synthesis (item #5)).	N/A
	13b	Describe any methods required to prepare the data for presentation or synthesis, such as handling of missing summary statistics, or data conversions.	N/A
	13c	Describe any methods used to tabulate or visually display results of individual studies and syntheses.	N/A
	13d	Describe any methods used to synthesize results and provide a rationale for the choice(s). If meta-analysis was performed, describe the model(s), method(s) to identify the presence and extent of statistical heterogeneity, and software package(s) used.	N/A
	13e	Describe any methods used to explore possible causes of heterogeneity among study results (e.g., subgroup analysis, meta-regression).	N/A
	13f	Describe any sensitivity analyses conducted to assess robustness of the synthesized results.	N/A
Reporting bias assessment	14	Describe any methods used to assess risk of bias due to missing results in a synthesis (arising from reporting biases).	N/A
Certainty assessment	15	Describe any methods used to assess certainty (or confidence) in the body of evidence for an outcome.	N/A
**RESULTS**			
Study selection	16a	Describe the results of the search and selection process, from the number of records identified in the search to the number of studies included in the review, ideally using a flow diagram.	136–142
	16b	Cite studies that might appear to meet the inclusion criteria, but which were excluded, and explain why they were excluded.	N/A
Study characteristics	17	Cite each included study and present its characteristics.	151–154
Risk of bias in studies	18	Present assessments of risk of bias for each included study.	405
Results of individual studies	19	For all outcomes, present, for each study: (a) summary statistics for each group (where appropriate) and (b) an effect estimate and its precision (e.g., confidence/credible interval), ideally using structured tables or plots.	143–147
Results of syntheses	20a	For each synthesis, briefly summarise the characteristics and risk of bias among contributing studies.	N/A
	20b	Present results of all statistical syntheses conducted. If meta-analysis was done, present for each the summary estimate and its precision (e.g., confidence/credible interval) and measures of statistical heterogeneity. If comparing groups, describe the direction of the effect.	212–217
	20c	Present results of all investigations of possible causes of heterogeneity among study results.	N/A
	20d	Present results of all sensitivity analyses conducted to assess the robustness of the synthesized results.	N/A
Reporting biases	21	Present assessments of risk of bias due to missing results (arising from reporting biases) for each synthesis assessed.	N/A
Certainty of evidence	22	Present assessments of certainty (or confidence) in the body of evidence for each outcome assessed.	N/A
**DISCUSSION**			
Discussion	23a	Provide a general interpretation of the results in the context of other evidence.	223–250
	23b	Discuss any limitations of the evidence included in the review.	N/A
	23c	Discuss any limitations of the review processes used.	276–282
	23d	Discuss implications of the results for practice, policy, and future research.	284–290
**OTHER INFORMATION**			
Registration and protocol	24a	Provide registration information for the review, including register name and registration number, or state that the review was not registered.	81–84
	24b	Indicate where the review protocol can be accessed, or state that a protocol was not prepared.	81–84
	24c	Describe and explain any amendments to information provided at registration or in the protocol.	81–84
Support	25	Describe sources of financial or non-financial support for the review, and the role of the funders or sponsors in the review.	297–302
Competing interests	26	Declare any competing interests of review authors.	307
Availability of data, code and other materials	27	Report which of the following are publicly available and where they can be found: template data collection forms; data extracted from included studies; data used for all analyses; analytic code; any other materials used in the review.	291–292; 306
